# Understanding Burnout Crossover Contagion: A Concept Analysis Using Walker and Avant’s Method

**DOI:** 10.1155/jonm/5313772

**Published:** 2026-10-17

**Authors:** Si-Jia Wang, Guang-Zhi Liu, Hong Dong, Hong-Lian Gao, Bin-Bin Zhang, Yu-Fang Guo

**Affiliations:** ^1^ School of Nursing and Rehabilitation Shandong University Jinan Shandong China sdu.edu.cn; ^2^ The Second Hospital of Shandong University Jinan Shandong China sdey.net; ^3^ Qilu Hospital of Shandong University Dezhou Hospital Dezhou Shandong China

**Keywords:** burnout contagion, burnout crossover, concept analysis, crossover of burnout, nursing management

## Abstract

**Aim(s):**

To clarify the concept of burnout crossover contagion using Walker and Avant’s concept analysis method and to identify its defining attributes, antecedents, consequences, and implications for nursing management.

**Background:**

Burnout may extend beyond individual employees through interpersonal processes within shared work environments. However, burnout crossover contagion remains inconsistently conceptualized, with unclear boundaries from related constructs and limited synthesis of its defining attributes, antecedents, and consequences.

**Evaluation:**

Walker and Avant’s concept analysis method was applied to synthesize formally included studies on burnout crossover and contagion. Evidence was examined to identify uses of the concept, defining attributes, antecedents, consequences, and empirical referents.

**Key Issue(s):**

Three defining attributes were identified: interpersonal interaction, direct and indirect transmission, and synchronization of burnout manifestations. Antecedents included shared occupational stressors, transmission‐related conditions, and individual characteristics, while consequences were identified at individual and organizational levels. A hypothesis‐generating nursing‐team‐specific extension was developed in which shiftwork, handoffs, staffing instability, task interdependence, and repeated team interaction are conceptualized as contextual or exposure‐related conditions that may increase opportunities for interpersonal burnout transmission rather than as established causal mechanisms.

**Conclusion(s):**

Burnout crossover contagion is an interpersonal phenomenon involving the transmission and increasing convergence of burnout manifestations between individuals. The proposed framework requires further empirical testing using longitudinal, dyadic, multilevel, and social network approaches, particularly to distinguish interpersonal transmission from burnout similarity attributable to shared occupational conditions.

**Implications for Nursing Management:**

Nurse managers should consider burnout as both an individual occupational health concern and a potential relational risk within nursing teams. Stable staffing, equitable workload allocation, sufficient recovery, supportive leadership, psychologically safe handoffs, and structured team support may represent potential management approaches; however, their effectiveness in preventing or reducing burnout crossover contagion requires empirical validation.

## 1. Background

Burnout is widely recognized as a multidimensional syndrome characterized by emotional exhaustion, cynicism, and diminished personal accomplishment, with emotional exhaustion often regarded as its core component [1]. While burnout is not a specific emotion, it is considered an emotion‐oriented construct, defined as “a response to emotional and interpersonal stressors in the workplace.” This distinction underscores that burnout is not merely a fleeting emotional issue but a cumulative response to persistent workplace challenges. The consequences of burnout extend beyond the individual, becoming a collective issue within organizations and threatening employees’ physical and mental well‐being [2, 3]. Burnout not only erodes individual productivity and satisfaction but may also extend to team members and be associated with poorer workplace climate and organizational functioning [4, 5]. Therefore, understanding the factors contributing to burnout and how it may propagate across individuals and teams is of critical importance.

Studies increasingly demonstrate that burnout‐related experiences can spread between individuals in workplace settings, including nurses and other healthcare professionals [6, 7]. Two key concepts, “contagion” and “crossover,” are particularly relevant to understanding this interpersonal process. In psychology, emotional contagion describes the tendency for individuals to converge emotionally through exposure to others’ emotional expressions, often involving relatively automatic processes such as mimicry and emotional synchronization [8]. In contrast, crossover, as conceptualized within crossover theory, refers to the interpersonal transmission of stress, strain, well‐being, or other psychological experiences from one individual to another within a shared social environment [9–11]. Although emotional contagion and crossover overlap, they differ in explanatory scope: emotional contagion primarily concerns the transmission of affective states, whereas crossover provides a broader framework for understanding the interpersonal transfer of psychological experiences, including burnout.

Other related perspectives further clarify the conceptual boundaries of burnout crossover contagion. Spillover differs from crossover because it refers to the transfer of experiences across domains within the same individual rather than between individuals [9, 12]. Collective burnout, meanwhile, describes a shared team‐level state and does not necessarily indicate interpersonal transmission, because similarity in burnout may also arise from common exposure to workload, staffing shortages, shiftwork, or other occupational stressors [6, 13]. Thus, shared burnout within a team should be distinguished from burnout that develops through interpersonal transmission. Burnout crossover contagion is therefore more specific than general crossover because it focuses on the interpersonal transmission and progressive convergence of burnout‐related emotions, attitudes, and behaviors [6, 14], while extending beyond emotional contagion by encompassing nonaffective and indirect interpersonal processes. Accordingly, crossover theory provides the primary theoretical lens for the present concept analysis, while emotional contagion and other interpersonal processes are considered potential mechanisms through which burnout may be transmitted.

In recent years, scholars have increasingly focused on the transmission effects of burnout, suggesting that individual burnout may affect spouses and other team members through crossover contagion [10]. For instance, individuals may observe and mimic their colleagues’ burnout‐related emotions and behaviors, treating their colleagues as “models” [15]. The reception of verbal and nonverbal feedback may also enable individuals to “catch” others’ emotions, while exposure to a colleague’s cynical attitudes or negative work behaviors can influence an individual’s own emotional and behavioral responses, potentially leading to diminished collaboration and impaired communication within the workplace. These effects ultimately undermine organizational performance and team dynamics [1, 16‐17].

Burnout crossover and burnout contagion represent related but distinct perspectives on interpersonal burnout transmission. Burnout crossover primarily emphasizes the interpersonal transmission of burnout from one individual to another, thereby highlighting a directional source–recipient relationship [9, 10]. Burnout contagion, in contrast, emphasizes the spread and increasing similarity of burnout manifestations through interpersonal interaction, observation, and communication [6, 15]. Thus, crossover primarily clarifies who influences whom, whereas contagion highlights how burnout manifestations may spread and become increasingly similar among interacting individuals. In the present study, the term burnout crossover contagion is used to integrate these complementary perspectives by capturing both directional interpersonal transmission and the convergence of burnout manifestations. It is therefore conceptualized as an integration of crossover and contagion perspectives rather than as an independent established theory.

Despite the growing body of studies on burnout crossover contagion, the concept remains insufficiently defined and its conceptual boundaries require further clarification. A mature concept requires clear definitions, well‐delineated attributes, and a thorough description of antecedents and consequences. Additionally, it should establish boundaries that allow for accurate measurement and practical application. The absence of these components hinders theoretical development and practical implementation [18]. Therefore, a systematic clarification of burnout crossover contagion is needed to establish its defining attributes, antecedents, consequences, and conceptual boundaries. This study adopts Walker and Avant’s concept analysis method to define the attributes of burnout crossover contagion and clarify its conceptual framework, aiming to provide a reference for future studies on this topic [19].

## 2. Methods

### 2.1. Design

The study employed Walker and Avant’s concept analysis method, a structured approach used to clarify a concept’s meaning, defining attributes, antecedents, consequences, and empirical referents [19]. Compared with evolutionary approaches that emphasize the dynamic and context‐dependent development of concepts, Walker and Avant’s method places greater emphasis on establishing clear conceptual boundaries and distinguishing a focal concept from related constructs [20, 21]. This approach was considered appropriate because burnout crossover contagion overlaps with emotional contagion, crossover, collective burnout, and the effects of shared occupational stressors.

The 2019 version of Walker and Avant’s concept analysis method was used because it was the most recent edition available when the study was designed and retained the established eight‐step process: selecting the concept, determining the purpose of the analysis, identifying its uses, determining defining attributes, constructing a model case and additional cases, identifying antecedents and consequences, and defining empirical referents [19]. This method provided a basis for clarifying burnout crossover contagion and developing a preliminary framework for future measurement and nursing management applications.

### 2.2. Search Strategy

The literature search was conducted across multiple Chinese and international databases, including China National Knowledge Infrastructure (CNKI), Wanfang Data, VIP Database, SinoMed, PubMed, the Cochrane Library, Web of Science, Embase, and CINAHL. Search terms in Chinese and English covered equivalent concepts, including “emotional exhaustion crossover,” “coworker burnout,” “interpersonal transmission of burnout,” “social contagion of burnout,” “burnout contagion,” “crossover of burnout,” “burnout crossover,” and “crossover of exhaustion.” Subject headings and free‐text terms were combined and adapted to the search requirements of each database. Reference lists of relevant articles were also manually screened to identify additional eligible studies. The search covered publications from database inception to September 2026.

### 2.3. Eligibility Criteria

Studies were eligible for inclusion if they: (1) examined burnout crossover, burnout contagion, or the interpersonal transmission of burnout‐related emotions, attitudes, or behaviors; (2) provided information relevant to the conceptual definition, uses, defining attributes, antecedents, consequences, transmission mechanisms, or empirical referents of burnout crossover contagion; (3) were conducted in workplace, organizational, educational, or other interpersonal contexts relevant to the concept; and (4) were published in Chinese or English.

Duplicate publications, editorials, letters, commentaries, book reviews, and articles without accessible full text were excluded. Studies focusing exclusively on the psychometric evaluation of an instrument, without providing information relevant to the meaning or interpersonal transmission of burnout, were also excluded.

### 2.4. Study Selection and Data Extraction

All records identified through the database searches were imported into EndNote for reference management and duplicate removal. A total of 4459 records were identified, and 662 duplicate records were removed. Subsequently, 3797 records were independently screened by two researchers according to the predefined eligibility criteria. After title and abstract screening, 3743 records were excluded, leaving 54 articles for full‐text assessment. Of these, 31 were excluded because they did not provide sufficient information for conceptual clarification, the full text was unavailable, or they were conference abstracts. Ultimately, 23 studies were included in the concept analysis. The study selection process is presented in Figure 1.

**FIGURE 1:**
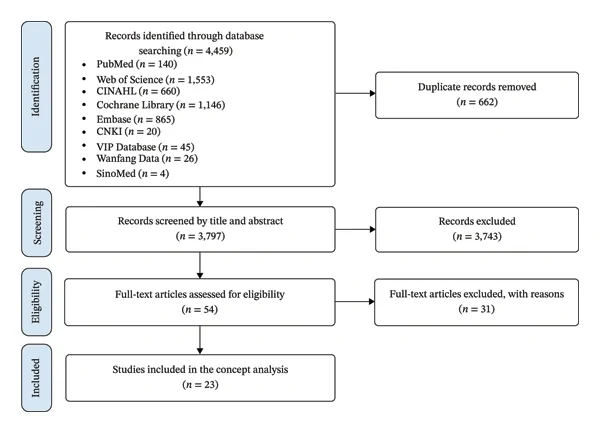
Flow diagram of study selection.

Disagreements were resolved through discussion or, when necessary, consultation with a third researcher. Using a structured data extraction form, two researchers independently extracted study characteristics, including authors, publication year, country or region, study design, participants, sample size, setting, relationship type, main findings, and conceptual contribution. Discrepancies were resolved through discussion or consultation with a third researcher.

### 2.5. Concept Analysis Procedure

Following Walker and Avant’s approach, the concept analysis was conducted using information extracted from the included studies. The literature was first examined to identify how burnout crossover contagion and related terms were defined and used across different occupational and relational contexts. Definitions, descriptions of interpersonal transmission, and reported manifestations of burnout crossover were compared across studies to identify recurring conceptual characteristics. These recurring characteristics were then synthesized to determine the defining attributes of burnout crossover contagion.

The extracted literature was subsequently examined to identify factors associated with the occurrence and development of the phenomenon. Conditions that preceded or facilitated burnout crossover contagion were categorized as antecedents, whereas outcomes occurring following interpersonal transmission were categorized as consequences. Particular attention was given to distinguishing interpersonal transmission from similarity in burnout arising from shared occupational stressors. Model, borderline, related, and contrary cases were then developed according to the presence or absence of the identified defining attributes to further clarify the conceptual boundaries of burnout crossover contagion. Finally, empirical referents were identified from the literature by examining how interpersonal exposure, transmission processes, and convergence in burnout manifestations had been observed or measured in previous studies. These findings were integrated to develop the conceptual framework and provide a basis for the preliminary measurement framework proposed in this study.

## 3. Results

### 3.1. Characteristics of Included Studies

A total of 23 studies published between 1999 and 2026 were included in the concept analysis, comprising nine cross‐sectional studies, 11 longitudinal studies, one experimental study, and two reviews. Detailed characteristics of the included studies, including country or region, study design, participants, sample size, setting, relationship type, main findings, and conceptual contribution, are presented in Supporting Table 1.

### 3.2. Methodological, Contextual, and Population Heterogeneity

The included studies showed substantial heterogeneity in study design, setting, and participant population. Methodological approaches included cross‐sectional, longitudinal, experimental, and review designs. The studies were conducted across a range of occupational and team‐based contexts, including healthcare, education, organizational, military, and sport settings, and involved nurses, physicians, teachers, managers, supervisors, subordinates, employees, and athletes. Most studies examined workplace‐based interpersonal relationships, including coworker, team‐member, and leader–follower relationships, whereas a smaller number were conducted in nontraditional team contexts. This diversity indicated that burnout crossover contagion had been examined across multiple relational and organizational contexts, while also limiting the direct comparability of findings across study designs, populations, and settings.

### 3.3. Uses of the Concept

Across the literature, burnout crossover contagion was used to describe the interpersonal spread of burnout‐related strain, emotions, attitudes, and behaviors across shared social environments. Early discussions suggested that one employee’s burnout could influence another, and subsequent work increasingly linked this process to emotional contagion and crossover perspectives [22‐23]. Later studies further conceptualized burnout crossover contagion as occurring through face‐to‐face interaction, in which employees could internalize colleagues’ cynical attitudes, emotional exhaustion, or negative work behaviors, thereby affecting their own emotions and responses [9, 24].

Subsequent empirical and theoretical work extended this understanding by emphasizing mechanisms such as mimicry, feedback, empathy, and interpersonal exposure. Burnout crossover contagion was gradually regarded as a phenomenon similar to emotional contagion, potentially involving both relatively automatic and more cognitively mediated processes [25‐26]. More recent studies also expanded the concept beyond peer‐level transmission, indicating that burnout could spread horizontally among colleagues as well as vertically between leaders and subordinates [27–29]. Overall, the concept was applied across service‐oriented and high‐interaction professions, including nurses, general practitioners, teachers, and police officers [6, 15, 30‐31]. However, despite its increasing use, burnout crossover contagion continued to overlap with related concepts such as emotional contagion and crossover, and no universally accepted definition had yet been established.

### 3.4. Defining Attributes

A critical step in concept analysis was the identification of defining attributes, which distinguished the focal concept from similar or related constructs. These attributes represented the essential characteristics consistently associated with the concept [19]. Despite variation in study design, occupational context, and population, three defining attributes consistently emerged across the literature: interpersonal interaction, direct and indirect transmission, and synchronization of burnout manifestations.

#### 3.4.1. Interpersonal Interaction

Burnout crossover contagion occurred through bidirectional emotional and psychological influences facilitated by communication, observation, and feedback among individuals [15]. These interactions included both explicit behaviors (e.g., verbal communication and body language) and implicit processes, such as emotional transfer and psychological contagion. Studies indicated that an individual’s burnout level was associated with that of their interaction partners. Frequent interpersonal interactions within a team were associated with similar emotional response patterns [26, 28]. For instance, when one employee experienced excessive work pressure, lacked motivation, or displayed emotional exhaustion, these negative states could spread through daily interactions, increasing the emotional burden on others and exacerbating burnout within the team.

#### 3.4.2. Direct and Indirect Transmission

Direct and indirect emotional contagion referred to the reactions individuals experienced when exposed to others’ emotional expressions, such as facial expressions, language, or behavior [15]. Burnout spread directly through the imitation of a colleague’s work attitude or behavior, or indirectly through perceiving others’ emotional states and generating empathy—both by understanding others’ emotions and partially synchronizing with them [6]. This process occurred either unconsciously, as individuals automatically responded to others’ emotions, or consciously, through deliberate efforts to interpret and understand others’ feelings [30]. When individuals imitated or resonated with the behaviors and emotions of those around them, they became more susceptible to the influence of others’ emotional states, resulting in shared emotional experiences. Within teams, when one member displayed signs of burnout, others could unconsciously mimic these emotions or perceive the burnout emotionally, leading to empathetic alignment and similar emotional responses.

#### 3.4.3. Synchronization of Burnout Manifestations

The synchronization of burnout manifestations constituted a core attribute ofburnout crossover contagion, referring to the alignment of burnout‐related behaviors and emotional states between individuals. Repeated emotional reinforcement among group members was suggested to contribute to convergence in emotional states and social cognition, aligning them with the emotional communicator. This phenomenon was particularly evident in high‐pressure work environments, especially during intensive team collaboration or when interaction partners shared similarities [14]. Studies suggested that individuals detected stress signals from others through verbal and nonverbal cues, activating processes such as empathy or priming effects within social interactions. Through repeated interpersonal exposure, burnout manifestations became increasingly similar among interacting individuals. Experimental and longitudinal evidence suggested that exposure to others’ burnout was followed by increases in corresponding burnout manifestations, including emotional exhaustion, cynicism or depersonalization, and reduced professional efficacy [14, 26]. Such convergence was distinguished from simultaneous high burnout levels arising solely from shared occupational stressors, because synchronization in burnout crossover contagion implied an interpersonal transmission process.

### 3.5. Antecedents

Antecedents were events or conditions that had to exist before a concept occurred.

#### 3.5.1. Shared Stressors

High‐pressure work environments constituted a contextual antecedent of burnout crossover contagion by increasing the likelihood that employees experienced burnout within the same work setting. High workload, time pressure, and demanding working conditions depleted employees’ emotional resources and increased vulnerability to exhaustion. High job demands were described as conditions that provided a “breeding ground” for burnout, particularly in demanding healthcare environments [10]. Workload and time pressure were similarly identified as occupational conditions associated with employees’ exhaustion [32].

Limited job resources were identified as the second contextual antecedent in this study. Based on previous literature, job resources can be broadly categorized into organizational resources, interpersonal/social resources, work‐organization resources, and task‐level resources [33]. In the reviewed literature, relevant resource‐related evidence was primarily identified in organizational and interpersonal/social resources. For organizational resources, insufficient administrative and organizational support was associated with greater vulnerability to burnout [32]. For interpersonal/social resources, colleagues experiencing higher burnout were less likely to provide individuals with sufficient resources, including knowledge, curricular materials, time, and emotional assistance, thereby facilitating burnout crossover contagion [27]. Within leader–follower relationships, leader burnout predicted reductions in followers’ job resources, including autonomy, social support, performance feedback, and professional development, which subsequently contributed to higher follower burnout [34]. These findings indicated that limited resources could increase individuals’ vulnerability to burnout crossover contagion.

#### 3.5.2. Transmission Pathways

Across the included studies, transmission‐related conditions primarily reflected two relational features. These included high interaction frequency, as well as relationship quality and mutual feedback among employees. These conditions shaped opportunities for interpersonal exposure and the extent to which individuals were receptive to others’ burnout manifestations.

Repeated exposure to others’ burnout manifestations, facilitated by high interaction frequency, was associated with a greater likelihood of individuals developing similar burnout manifestations. Perceived colleague burnout was more strongly associated with emotional exhaustion and depersonalization when work‐related problems were discussed frequently [15]. Stronger patterns of burnout crossover contagion were similarly observed within frequent, multiplex, and embedded interpersonal ties, although longitudinal findings varied across burnout dimensions [26]. In supervisor–subordinate relationships, supervisors’ emotional exhaustion predicted subsequent counselors’ emotional exhaustion when perceived interaction frequency was high [35]. More recent evidence showed that communication‐based exposure to coworkers’ burnout facilitated burnout crossover contagion [36]. These findings indicated that high interaction frequency was an important transmission‐related condition for burnout crossover contagion among employees.

Relationship quality and mutual feedback further conditioned whether repeated exposure to burnout could develop into burnout crossover contagion. A study found that dentists’ exhaustion was associated with dental nurses’ exhaustion when interactions were frequent and the dentist was perceived as friendly, or when mutual feedback was high [8]. These findings indicated that relationship quality and mutual feedback functioned as relational boundary conditions associated with the likelihood of burnout crossover contagion.

#### 3.5.3. Individual Characteristics

Individual characteristics associated with burnout crossover contagion included emotional susceptibility [15, 24, 30], self‐efficacy [28‐29], and occupational characteristics [14, 37].

Emotional susceptibility appeared to be an important individual condition influencing burnout crossover contagion. High levels of susceptibility to emotional contagion were associated with particularly high levels of emotional exhaustion and depersonalization [15]. In supervisor–subordinate relationships, subordinate susceptibility to emotional contagion strengthened the associations between supervisors’ and subordinates’ corresponding burnout manifestations [24]. These findings suggested that individuals who were more emotionally responsive to others might be more vulnerable to burnout crossover contagion.

Self‐efficacy represented another individual characteristic associated with burnout crossover contagion. In top–down relationships, lower levels of employee self‐efficacy strengthened the crossover of burnout from managers to employees, whereas higher self‐efficacy buffered this effect [28]. In bottom–up relationships, higher leader self‐efficacy strengthened burnout crossover contagion from followers to leaders [29], possibly because leaders with higher emotional self‐efficacy paid greater attention to followers’ emotional states and were therefore more susceptible to their negative emotions. These findings indicated that self‐efficacy functioned as a context‐dependent boundary condition for burnout crossover contagion.

Selected occupational characteristics could further condition burnout crossover contagion. The indirect crossover of emotional exhaustion through increased hindrance stressors was stronger among employees with shorter organizational tenure [37]. Experimental evidence also showed that cynicism crossover was stronger when recipients were exposed to a burned‐out colleague of similar occupational rank [14]. These findings suggested that occupational experience and source–recipient occupational similarity might influence the strength of burnout crossover contagion.

Potential influencing factors were also reported, including gender, work experience, and socioeconomic context [32]. However, these factors were not included as established antecedents because the available studies did not directly examine their effects on burnout crossover contagion.

### 3.6. Consequences

Consequences were the outcomes resulting from the occurrence of a concept.

#### 3.6.1. Individual Consequences

At the individual level, burnout crossover contagion was associated with increased burnout manifestations and reduced professional efficacy among employees. Increased burnout manifestations were reflected primarily in greater emotional exhaustion, depersonalization, and cynicism, whereas reduced professional efficacy reflected a decline in employees’ perceived effectiveness at work [6, 14, 26]. Across coworker, supervisor–subordinate, and team‐based relationships, exposure to others’ burnout was associated with greater emotional exhaustion, depersonalization or cynicism, and reduced professional efficacy [6, 14, 24, 26]. Experimental evidence further showed that exposure to a burned‐out colleague increased cynicism and reduced professional efficacy, indicating that interpersonal burnout exposure adversely affected occupational attitudes and perceived professional efficacy [14]. Longitudinal and network‐based evidence also demonstrated that the effects of burnout crossover contagion persisted over time, particularly for emotional exhaustion [26]. These findings indicated that increased burnout manifestations and reduced professional efficacy were individual consequences of burnout crossover contagion.

#### 3.6.2. Organizational Consequences

At the organizational level, burnout crossover contagion was associated with a greater team‐level burnout burden. Burnout spread continuously through interactions among employees and accumulated over time, thereby creating an organizational‐level burnout climate and burden [6]. Social‐network evidence further indicated that burnout spread through interpersonal ties among interconnected employees, thereby increasing the overall burnout burden within the organization.

Another organizational consequence was declining organizational performance. Employees’ burnout was described as hampering task performance and making organizational strategic and operational objectives more difficult to achieve, thereby adversely affecting organizational performance [28]. Further empirical evidence showed that coworker burnout increased employees’ own burnout, which subsequently reduced their work performance and impaired team performance, thereby adversely affecting organizational performance [38]. Taken together, these findings supported declining organizational performance as an organizational consequence of burnout crossover contagion.

### 3.7. Constructing Cases

Cases could be derived from the literature, real‐life situations, or hypothetical examples. In this study, no suitable real cases were identified during the literature review. Therefore, the model, borderline, and contrary cases were constructed based on the results of the concept analysis. The model case included all defining attributes of burnout crossover contagion, the borderline case included only some of them, and the contrary case included none.

#### 3.7.1. Model Case

This example illustrated all the defining attributes of burnout crossover contagion.

During an accreditation review at a tertiary hospital, the emergency department nursing team faced significant workload pressures, strict evaluation standards, and extended overtime hours. Nurse Xiao Li began to exhibit pronounced emotional exhaustion and stress due to the prolonged intensity of her work. Through frequent workplace interactions, Xiao Wang observed Xiao Li’s low mood, procrastination, and emotional exhaustion. He empathized with her situation and resonated with her stress. As Xiao Li’s emotional exhaustion worsened, Xiao Wang gradually experienced increasing work‐related strain and ultimately developed similar symptoms of emotional exhaustion.

#### 3.7.2. Borderline Case

A borderline case differed from a model case in that it did not exhibit all defining attributes of the concept.

As the hospital prepared for its accreditation review, Nurse Xiao Li displayed symptoms of emotional exhaustion, reduced focus, and task procrastination due to consecutive high‐intensity workdays. Her colleague, Xiao Wang, who worked closely with her, noticed her negative mood and fatigue through frequent interactions. However, Xiao Wang’s extensive work experience and strong coping skills enabled him to effectively regulate his emotions and manage stress, preventing him from developing noticeable burnout manifestations.

#### 3.7.3. Contrary Case

A contrary case lacked all defining attributes of burnout crossover contagion.

Head Nurse Xiao Guo experienced stress recently while applying for a senior professional title. However, she managed to alleviate her stress through mindfulness and meditation. At work, Xiao Guo did not display significant signs of emotional exhaustion, nor did she openly discuss her stress with colleagues. The medical team members interacted infrequently and focused on their own tasks, leaving Xiao Guo’s emotional state largely unnoticed. As a result, her stress did not affect her colleagues, and the team maintained a positive working atmosphere without any signs of burnout transmission.

### 3.8. Empirical Referents

The final step in concept analysis involved identifying empirical referents for the attributes of the concept. These referents aided in measuring the concept and provided further clarification.

One method for assessing burnout crossover contagion was collective burnout measurement, which adapted the Maslach Burnout Inventory‐General Survey (MBI‐GS) using the referent‐shift consensus model [13]. This approach shifted the focus from individual experiences to group‐level perceptions. For instance, items such as “I feel emotionally exhausted at the end of a workday” were rephrased as “My colleagues feel emotionally exhausted at the end of a workday.” Such adjustments allowed researchers to assess how employees perceived burnout within their teams.

Another approach involved evaluating perceived colleague burnout, focusing on how employees viewed the burnout levels of their coworkers [6, 15, 30]. This approach was operationalized using a small number of items assessing employees’ perceptions of colleague burnout. Example items included: “How many of your colleagues do you think are experiencing burnout?” and “How many of your colleagues feel emotionally exhausted due to their work?” These items were rated on a scale from 1 (none) to 5 (all colleagues), and responses were aggregated into a single index reflecting perceived burnout among colleagues.

Although existing approaches provided preliminary ways to assess perceived burnout in teams or among colleagues, they still lacked the capacity to systematically capture the dynamic and interpersonal nature of burnout crossover contagion. Thus, there remained a need to develop new tools that could scientifically and systematically measure burnout crossover contagion.

### 3.9. Working Definition

In summary, burnout crossover contagion refers to a phenomenon in which, within a shared organizational environment, one individual’s burnout is transmitted to others through direct or indirect interpersonal processes under the influence of shared stressors, transmission‐related conditions, and individual characteristics, thereby promoting convergence in burnout manifestations among group members.

### 3.10. Conceptual Framework

Based on the literature synthesis, a conceptual framework was developed to integrate the antecedents, defining attributes, and consequences of burnout crossover contagion (Figure 2). The antecedents include shared occupational stressors, transmission‐related conditions, and individual characteristics, which may shape the likelihood and strength of interpersonal interaction, direct and indirect transmission, and synchronization of burnout manifestations. An evidence mapping of the formally included studies to the identified antecedents, defining attributes, and consequences is provided in Supporting Table 3.

**FIGURE 2:**
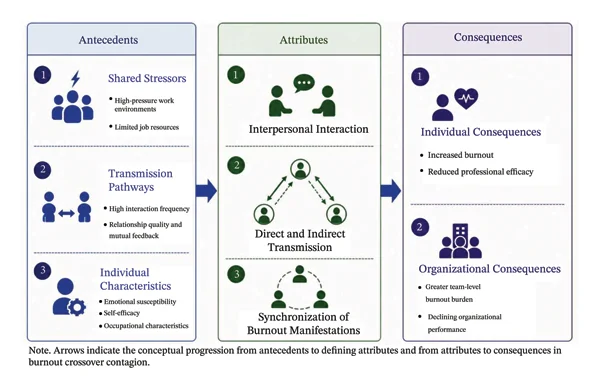
Conceptual diagram of burnout crossover contagion.

Building on this general framework, a hypothesis‐generating nursing‐team‐specific extension was developed (Figure 3). Nursing‐specific occupational conditions, such as staffing instability, high workload, and shiftwork, may increase nurses’ vulnerability to burnout, while exposure‐related conditions, such as handoffs, task interdependence, and repeated team interaction, may increase opportunities for interpersonal exposure and repeated contact among nurses. Together, these conditions may create a context in which burnout crossover contagion is more likely to occur. Under such conditions, repeated exposure to others’ burnout may contribute to increasing similarity in burnout manifestations among team members. Potential nursing‐specific downstream implications were considered to extend to team functioning, organizational stability, care quality, and patient safety. Because these nursing‐specific relationships have not yet been directly established, the model is intended to generate testable hypotheses rather than to imply causal pathways. The nursing‐specific model therefore represents a contextual extension rather than a redefinition of the general concept.

**FIGURE 3:**
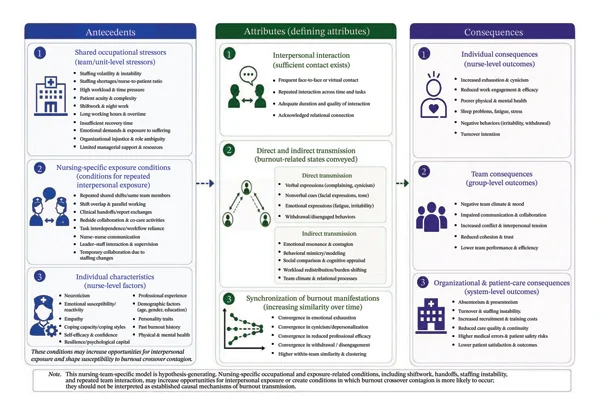
Hypothesis‐generating nursing‐team‐specific extension of the conceptual model of burnout crossover contagion.

### 3.11. Preliminary Measurement Framework

Based on the antecedents, defining attributes, consequences, and empirical referents identified through the concept analysis, a preliminary measurement framework was developed to guide the future operationalization of burnout crossover contagion among nurses (Supporting Table 2). The framework emphasizes linked assessment of interacting individuals, interpersonal exposure, direct and indirect transmission processes, and changes in burnout manifestations over time. The three defining attributes—interpersonal interaction, direct and indirect transmission, and synchronization of burnout manifestations—constitute the core measurement domains, while shared occupational stressors, transmission‐related conditions, individual characteristics, and downstream outcomes serve as supporting domains. Shared occupational stressors should also be assessed to distinguish interpersonal crossover from burnout similarity arising from common working conditions. Testable propositions were formulated to guide future longitudinal, dyadic, multilevel, and social network studies. The proposed measurement domains and propositions are hypothesis‐generating and require empirical validation before they can be regarded as established measures of burnout crossover contagion.

## 4. Discussion

This concept analysis clarifies burnout crossover contagion as a complex interpersonal and organizational phenomenon rather than a simple accumulation of individual burnout. By synthesizing its uses in the literature, defining attributes, antecedents, consequences, and empirical referents, this study provides a clearer conceptual foundation for understanding how burnout manifestations may be transmitted across individuals within shared work environments.

Grounded in crossover theory, burnout crossover contagion retains the theory’s central emphasis on the interpersonal transfer of psychological experiences between individuals. This perspective allows burnout transmission to be understood as a relational and directional process that may occur between colleagues and across leader–employee relationships, while also recognizing that the strength of crossover may depend on interpersonal exposure, relationship characteristics, and individual susceptibility [9, 10, 28, 29, 34]. Emotional contagion and related interpersonal processes further help explain how such crossover may occur, including through emotional resonance, mimicry, empathy, and social comparison [6, 14‐15, 30]. By integrating the directional emphasis of crossover with the spreading and convergence emphasized by contagion, burnout crossover contagion provides a focused conceptual framework for understanding the interpersonal transmission and synchronization of burnout manifestations within shared work environments.

A key contribution of this concept analysis is the identification of three defining attributes: interpersonal interaction, direct and indirect transmission, and synchronization of burnout manifestations. Together, these attributes distinguish burnout crossover contagion from the mere coexistence of burnout among individuals and emphasize its interpersonal and dynamic nature. In particular, synchronization suggests that repeated interpersonal exposure may be reflected in increasing similarity in burnout manifestations over time rather than simply simultaneous high burnout levels.

The literature included in this analysis was heterogeneous in design, context, and population. Most studies were observational and were conducted in service‐oriented or high‐interaction professions, including healthcare, education, and other organizational settings. This heterogeneity broadens the potential applicability of the concept but also suggests that the mechanisms, intensity, and manifestations of burnout crossover contagion may vary across occupational and relational contexts. Findings from one setting should therefore not be generalized uncritically to another.

In nursing teams, burnout crossover contagion may occur horizontally among colleagues and vertically across leader–staff relationships. Shared occupational stressors, such as high workload, staffing instability, shiftwork, insufficient recovery, and limited organizational support, may increase vulnerability to burnout but do not themselves indicate interpersonal transmission [1, 39–41]. Repeated shared shifts, clinical handoffs, task interdependence, and frequent interpersonal interactions may instead increase opportunities for exposure to others’ burnout manifestations. Evidence of burnout crossover contagion suggests that such exposure may facilitate interpersonal transmission under certain relational and individual conditions [6, 29, 34]. Emotional susceptibility and self‐efficacy may further shape responsiveness to such exposure [28]. The proposed nursing‐specific model should therefore be interpreted as hypothesis‐generating, with the relationships among these factors representing proposed pathways rather than established causal mechanisms.

Through repeated interpersonal interaction, burnout manifestations may be transmitted directly through verbal expressions, nonverbal cues, emotional displays, and withdrawal behaviors, or indirectly through processes such as emotional contagion, behavioral mimicry, empathy, social comparison, and cognitive appraisal [7, 14‐15]. With continued exposure, interacting team members may become increasingly similar in emotional exhaustion, cynicism or depersonalization, and reduced professional efficacy, reflecting synchronization of burnout manifestations. This differs from similar burnout levels arising solely from shared occupational stressors because burnout crossover contagion implies an interpersonal exposure and transmission process rather than simple co‐occurrence [13].

The most directly supported consequences of burnout crossover contagion concern recipients’ increased burnout manifestations, reduced professional efficacy, greater team‐level burnout burden, and impaired work performance. Broader effects on team climate, communication, collaboration, staffing stability, care quality, and patient safety are theoretically plausible but remain less directly established by crossover‐specific evidence. These outcomes should therefore be regarded as potential downstream implications requiring further empirical investigation rather than established core consequences of burnout crossover contagion.

Finally, this analysis highlights the need to move beyond isolated individual or aggregate burnout measures when examining burnout crossover contagion. Future studies should capture the interpersonal and temporal nature of the phenomenon by linking burnout levels across interacting individuals, assessing interpersonal exposure and potential transmission processes, and examining changes in burnout manifestations over time while accounting for shared occupational stressors. Longitudinal dyadic, multilevel, and social network designs may therefore be particularly useful for empirically testing the proposed concept, the hypothesis‐generating nursing‐specific extension, and the preliminary measurement framework. These proposed relationships and measurement domains require empirical validation before they can be regarded as established explanatory or measurement models.

### 4.1. Limitations

This study reviewed a limited number of Chinese and English databases and included only articles meeting specific inclusion criteria. Consequently, relevant literature may have been overlooked. In addition, the included studies were heterogeneous in methodology, context, population, and cultural background, which may have affected the comparability of the evidence and the interpretation of the conceptual synthesis. Most existing research on burnout crossover contagion is observational and focuses on specific occupational groups, without exploring causal relationships in depth. This study’s reliance on Walker and Avant’s concept analysis method may not fully capture the historical development of the concept, which may limit the comprehensiveness and analytical depth of the findings.

## 5. Conclusion

This concept analysis clarifies burnout crossover contagion as a distinct interpersonal and dynamic phenomenon characterized by interpersonal interaction, direct and indirect transmission, and synchronization of burnout manifestations. By identifying its antecedents, consequences, and empirical referents, the analysis further distinguishes burnout crossover contagion from the simple co‐occurrence of burnout arising from shared occupational stressors. Building on crossover theory, a nursing‐team‐specific hypothesis‐generating extension of the conceptual model was developed to illustrate how occupational stressors, repeated interpersonal exposure, and individual susceptibility may create conditions for burnout transmission within nursing teams. A preliminary measurement framework and testable propositions were also proposed to support the future operationalization and empirical examination of the concept.

Future studies should validate and refine the proposed measurement framework and examine the temporal and interpersonal processes underlying burnout crossover contagion using longitudinal, dyadic, multilevel, social network, experimental, and qualitative approaches. Greater attention should also be given to distinguishing interpersonal transmission from shared environmental effects and to evaluating context‐specific strategies for preventing or reducing burnout crossover contagion in nursing teams.

### 5.1. Implications for Nursing Management

Burnout crossover contagion has important implications for nursing management because burnout may represent not only an individual occupational health concern but also a relational risk within nursing teams. Rather than focusing on individual nurses as potential “sources” of burnout, nurse managers should consider team emotional climate, patterns of interpersonal exposure, handoff communication, leader–staff relationships, and team‐level patterns of burnout manifestations when identifying potential team‐level risks. Early identification should be accompanied by organizational and team‐level measures that address shared occupational stressors and support constructive team interactions [42].

Potential management approaches include adequate and stable staffing, equitable workload allocation, fair scheduling, sufficient recovery between shifts, supportive leadership, psychologically safe handoffs, structured team debriefing, peer support, and accessible psychological services [43‐44]. Particular attention may be warranted during periods of staffing instability, repeated shift overlap, high workload, or frequent handoffs, when opportunities for repeated interpersonal exposure may increase. Nurse leaders should also model constructive communication and emotional regulation and foster an environment in which staff can discuss workload and emotional demands without fear of blame [43].

Individual‐level approaches, such as stress‐management, resilience‐building, or mindfulness programs, may provide additional support [45], but should complement rather than replace organizational and team‐level strategies. Because these management approaches are conceptually derived from the present analysis, they should be regarded as preliminary and require empirical validation before their effectiveness in preventing or reducing burnout crossover contagion can be established.

## Author Contributions

Si‐Jia Wang: writing–review and editing, writing–original draft, visualization, validation, supervision, methodology, data curation, and conceptualization. Guang‐Zhi Liu: conceptualization, methodology, formal analysis, visualization, and writing–review and editing. Hong Dong: writing–review and editing, validation, supervision, methodology, investigation, and data curation. Hong‐Lian Gao: writing–review and editing, methodology, and formal analysis. Bin‐Bin Zhang: writing–review and editing, methodology, and investigation. Yu‐Fang Guo: writing–review and editing, writing–original draft, validation, supervision, project administration, methodology, investigation, and formal analysis.

## Funding

This study was funded by Shandong Province Research Project on Health Human Resource Management (2025RLZY065).

## Ethics Statement

The authors have nothing to report.

## Conflicts of Interest

The authors declare no conflicts of interest.

## Supporting Information

Additional supporting information can be found online in the Supporting Information section.

## Supporting information


**Supporting Information** Supporting Table 1. Articles included in the concept analysis. Supporting Table 2. Preliminary measurement framework and testable propositions for burnout crossover contagion. Supporting Table 3. Evidence mapping of formally included studies to conceptual elements of burnout crossover contagion.
