# Supplementary material for: Understanding Burnout Crossover Contagion: A Concept Analysis Using Walker and Avant’s Method
Source: J Nurs Manag. 2026 Oct 17;2026:5313772. doi: 10.1155/jonm/5313772 (PMC13675832; doi:10.1155/jonm/5313772)
Supplement: Supplementary file 1 [file JONM-2026-5313772-s001.docx]

**Supplementary Table 1. Articles included in the concept analysis**

|  | **Authors (Year)** | **Country/Region** | **Study Design** | **Participants** | **Sample Size** | **Setting** | **Relationship Type** | **Main Results** | **Concept** |
| --- | --- | --- | --- | --- | --- | --- | --- | --- | --- |
| 1 | Parent-Lamarche & Fernet (2020) | Québec, Canada | Longitudinal study | School principals and teachers | 76 principals; 102 teachers | Elementary and secondary schools; principal–teacher relationships | Principal–teacher; top-down | An indirect top-down crossover effect was found for emotional exhaustion only, suggesting that managerial emotional exhaustion may spread to employees with low self-efficacy. | Crossover refers to an interpersonal process where stress or its psychological effects transfer between individuals within a shared environment. This can occur directly through empathy, indirectly via moderating variables, or due to shared stressors like economic issues or work demands. |
| 2 | Hakanen, Perhoniemi, & Bakker (2014) | Finland | Cross-sectional study | Dentists and dental nurses | 470 dentist–dental nurse dyads | Dental healthcare; dentist–dental nurse working dyads | Dentist–dental nurse; mainly top-down | Exhaustion may cross over particularly from exhausted dentists (leading position) to dental nurses (subordinate position) under conditions of positive and frequent collaboration. | Job strain may cross over from one individual to another through direct crossover, indirect crossover, or shared stressors. In work dyads, this process is shaped by interpersonal interaction and hierarchical relationships. |
| 3 | González-Morales et al. (2012) | Spain | Two-wave panel study | Teachers | 555 teachers in 100 schools | Primary and secondary schools | School/collective climate–individual teacher | Perceived collective burnout at Time 1 was a significant predictor of burnout at Time 2 after considering previous levels of burnout,demands (workload, teacher student ratio, and absenteeism rates), and resources (quality of school facilities). | Perceived collective burnout refers to employees’ shared perceptions of how burned out their colleagues are, and it may predict individual burnout even without direct contagion or close interpersonal contact. |
| 4 | Bakker & Schaufeli (2000) | The Netherlands | Cross-sectional study | High-school teachers | 154 teachers | Dutch high schools; interactions among teaching colleagues | Teacher–teacher; coworker–coworker | The prevalence of perceived burnout among participants’ colleagues was most strongly related to individual teachers’ burnout (i.e., emotional exhaustion and depersonalization), when the teachers were highly susceptible to the emotions of others and when they frequently communicated with each other about work-related problems. | Colleagues may act as role models whose symptoms are imitated through a process of emotional contagion. That is, individuals under stress may perceive symptoms of burnout in their colleagues and automatically take on these symptoms. |
| 5 | Westman et al. (2011) | The Netherlands | Longitudinal study | Employees in work teams | 310 employees in 100 teams | Employment-agency branches; small interdependent work teams | Team–individual member | Crossover of job demands and emotional exhaustion across time from the group to individual team members only in teams characterized by high levels of cohesiveness and social support. | The interpersonal process that occurs when job stressors or strain experienced by one person affect the level of stressors or strain of another person in the same social environment is referred to as crossover. Crossover is an inter-individual transmission of stress or strain. A similar process is emotional contagion defined as the tendency to automatically mimic and synchronize expressions, feelings, and attitudes with those of another person and, consequently, to converge emotionally. |
| 6 | Huang et al. (2016) | China | Longitudinal study | Leaders and followers | 104 leader–follower dyads | Software-development, electronic-engineering and agricultural-product companies | Leader–follower; top-down | Controlling for follower burnout at T1, leader burnout at T1 is negatively related to changes of followers’ job and personal resources over a period of 6 months, which in turn are negatively related to follower burnout at T2. | Crossover refers to the process whereby job stress or psychological strains are transmitted inter-individually. Westman and Etzion (1999) have considered the crossover of burnout among close individuals as an indirect process mediated by interpersonal interactions. |
| 7 | Westman & Bakker (2008) | International | Review article | Healthcare professionals, employees, teams, and working couples represented in reviewed studies | Not applicable; narrative review | Healthcare organizations, hospitals, ICUs, medical teams, occupational groups and work–family contexts | Coworker–coworker; leader–follower; employee–spouse; team–member | Burnout is contagious and doctors and nurses transfer their burnout and other psychological strains to their team members with whom they interact. | Crossover refers to the interpersonal process in which job stress or psychological strain experienced by one person affects the strain of another person in the same social environment. Burnout is considered contagious and may be transferred to team members through workplace interaction. |
| 8 | Bakker et al. (2001) | The Netherlands | Cross-sectional study | General practitioners | 507 general practitioners | General medical practices; collaborating general practitioners | General practitioner–general practitioner; coworker–coworker | Perceived burnout complaints among colleagues, and individual differences in the susceptibility to emotional contagion are positively associated with emotional exhaustion. | Human service professionals may “catch” the negative feelings, the cynical attitudes, or the impaired job behaviors of their colleagues through the conscious or unconscious induction of emotional states and behavioral attitudes. |
| 9 | Zimmerman (2018) | United States | Review article | Preservice, early-career, and experienced teachers discussed; no original sample | Not applicable | Teacher-education programs and school organizational climates, especially the transition into early-career teaching | Teacher–teacher; school climate–teacher | Burnout can be contagious, and, therefore, I argue that teacher educators must expand the scope of their efforts to  include not only educating individual pre-service teachers but also revitalizing the organizational climates present within local schools. | Burnout contagion refers to the transmission of negative affect, such as emotional exhaustion and cynicism, from one person to another within a shared environment, and organizational climate is an important context for this process. |
| 10 | Bakker, Le Blanc, & Schaufeli (2005) | 12 European countries | Cross-sectional study | ICU nurses | 1,849 nurses in 80 ICUs | Hospital intensive care units | Nurse–nurse; coworker–coworker; unit-level | Analysis of variance indicated that the between-unit variance on a measure  of perceived burnout complaints among colleagues was statistically significant and substantially larger than the within-unit variance. The results of multilevel analyses showed that burnout complaints among colleagues in intensive care units made a statistically significant and unique contribution to explaining variance in individual nurses’ and whole  units’ experiences of burnout, i.e. emotional exhaustion, depersonalization and reduced personal accomplishment. Moreover, for both emotional exhaustion and depersonalization, perceived burnout complaints among colleagues was the most important predictor of burnout at the individual and unit levels, even after controlling for the impact of well-known organizational stressors as conceptualized in the demand-control model. | Burnout is contagious and may cross over from one nurse to another within intensive care units, where colleagues’ burnout complaints reflect the interpersonal presence of burnout in the work environment. |
| 11 | Meredith et al. (2020) | Country not clearly reported in the previously extracted manuscript text | Social network study with cross-sectional and longitudinal analyses | Secondary-school teachers | 931 teachers in 14 schools cross-sectionally; 578 teachers in 12 schools longitudinally | Secondary-school teacher teams | Teacher–teacher social-network ties | Burnout contagion was more pronounced when relationships were strong in terms of frequency, embeddedness, and multiplexity. The results also showed that features of relationships played a differential role in the contagion of different components of burnout. | Burnout contagion is regarded as a socially induced phenomenon in which interpersonal interactions act as conduits for the transmission of burnout, and the strength of these relationships shapes the contagion process. |
| 12 | Chullen (2014a) | United States | Cross-sectional study | Supervisors and subordinates | 93 supervisor–subordinate dyads | Mental-health services organization; supervisor–subordinate dyads | Supervisor–subordinate; top-down | Supervisor depersonalization was positively related to subordinate depersonalization and negatively related to subordinate perceptions of leader–member exchange. In addition, subordinate perceptions of leader–member exchange were negatively related to subordinate depersonalization and partially mediated the relationship between supervisor depersonalization and subordinate depersonalization. | Burnout may spread within supervisor-subordinate dyads through emotional contagion, and the supervisor-subordinate relationship (leader-member exchange) may serve as an important interpersonal pathway in this process. |
| 13 | Chullen (2014) | United States | Cross-sectional study | Supervisors and subordinates | 103 supervisor–subordinate dyads | Supervisor–subordinate relationships | Supervisor–subordinate; top-down | Supervisor burnout influences  subordinate burnout via the emotional contagion process and that subordinate susceptibility to emotional contagion  serves as an important individual difference variable that moderates this relationship. | Supervisor burnout may influence subordinate burnout through emotional contagion, and susceptibility to emotional contagion is treated as an important individual difference in this process. |
| 14 | Kim et al. (2017) | United States; Michigan and Indiana | Longitudinal study | Early-career teachers, mentors, and close colleagues | 171 early-career teachers from 10 districts; nominated mentors and colleagues also surveyed | Elementary and middle schools; early-career teachers, formal mentors and close colleagues | Mentor/close colleague–early-career teacher; school–teacher | The strongest association was found between ECTs' organizational exposure to burnout and their burnout levels at time 2 (i.e., their burnout levels at the end of the school year). Although ECTs who worked at schools serving mostly low-SES students tended to be more burned out, after including organizational exposure to burnout in the model, students' SES levels no longer had a significant effect on ECTs' burnout. Although the magnitude was weaker, ECTs' social network exposure to burnout also had a significant positive impact on their burnout levels at time 2 even after controlling for ECTs' burnout  level at time 1. compared to formal mentors, close colleagues that ECTs sought out for assistance had more influence on their burnout levels. | Burnout contagion may be understood through emotional contagion and cognitive “tuning in” to others’ emotions. In this study, social network exposure and organizational exposure were treated as two different forms of exposure to others’ burnout. |
| 15 | Vande Griek (2019) | United States | Longitudinal study | Counselors and supervisors | 290 counselor–supervisor dyads; 290 counselors and 121 supervisors in 26 organizations | Inpatient and outpatient substance-abuse treatment organizations; counselor–supervisor dyads | Supervisor–subordinate; upward and downward | No support was found for direct spread of emotional exhaustion across supervisor–subordinate dyads over time in the primary model. However, subordinate neuroticism and perceived frequency of interaction moderated the path from supervisor emotional exhaustion at T1 to subordinate emotional exhaustion at T2. | Burnout contagion is not the spread of an emotion per se, but the spread of an emotionally oriented construct characterized by emotional exhaustion, depersonalization, and reduced personal accomplishment, examined through the lenses of emotional contagion and crossover. |
| 16 | Järvinen (2021) | Finland | Cross-sectional study | Teachers | 1,531 teachers | Primary, lower-secondary and combined comprehensive schools across six geographical areas | Potential teacher–teacher/community crossover; not directly measured | Many participants reported varying levels of burnout, and the burnout symptoms were positively correlated with one another. Based on the research findings it can be suggested that individual attributes, including male gender and higher number of years in the profession, buffer from the crossover of burnout. In addition, the higher socio-economic status (SES) of the school neighborhood and smaller school size also act as buffers. On the other hand, exposing attributes include the female gender, less years in the profession, lower socio-economic status of the school neighborhood and large school size. | Burnout contagion is regarded as a form of emotional contagion that occurs through interpersonal interaction in the same social environment. Crossover refers to the transmission of stress or strain from one person to another through regular interaction. |
| 17 | Bakker, Westman, & Schaufeli (2007) | The Netherlands | Experimental study | Teachers and soldiers | Study 1: 40 teachers; Study 2: 101 soldiers | Secondary schools and a Dutch military battalion | Stimulus colleague–participant; peer/similar-rank source | Participants’ burnout (exhaustion and  depersonalization) was higher in the burnout condition compared to the  control condition. The results confirmed the crossover of burnout (cynicism and reduced professional efficacy). In line with predictions, a significant interaction effect for cynicism revealed that the crossover of burnout is moderated by similarity with the stimulus person. | The process that occurs when psychological strain experienced by one person affects the level of strain of another person who shares the same social environment is referred to as crossover. Crossover is an interindividual transmission of stress and strain. |
| 18 | Dong Fuping (2017) | China; northern Anhui Province | Cross-sectional study | Author-defined ‘special female faculty and staff’ groups | 500 female faculty and staff in six special-circumstance categories | Fuyang Vocational and Technical College, Fuyang Teachers College and Huaibei Normal University; informal groups of “special female faculty and staff” | Coworker–coworker within informal subgroups | Even if the influence of one's own work stress is eliminated, the influence of colleague burnout will still have a significant effect on the three burnout dimensions. | Occupational burnout contagion refers to the process by which individuals are affected by others’ low mood and negative work attitudes, allowing burnout to spread and accumulate within a work group. |
| 19 | Westman & Etzion (1999) | Israel | Cross-sectional study | School principals and teachers | 47 principals and 183 teachers in 47 schools | Elementary schools; principal–teacher hierarchical relationships | Principal–teacher; bidirectional | Principals’ job stress predicted their burnout, but the direct crossover of burnout from principals to teachers and from teachers to principals was not supported. | The process that operates when a stressor or psychological strain experienced by one person affects the level of strain of another person in the same social environment is referred to in the literature as crossover. A person's stress generated at the workplace can cross over to other job incumbents with whom she or he interacts at work. Individuals in the work team who share the same environment may start a crossover chain of stress and strain among themselves, whether the source of stress is in the families or at the workplace. |
| 20 | Wirtz et al. (2016) | Germany and Sweden | Longitudinal study | Team members and direct leaders | 315 team members under 67 leaders | Teams in banking, auditing, social services, education and facility management | Follower/team–leader; upward | Multiple regression analyses revealed that followers’ work engagement was positively related to leaders’ work engagement eight months later, controlling for followers’ tenure with the leader, leader gender, autonomy, workload, and work engagement at Time 1. Leaders’ emotional self-efficacy did not moderate the crossover of work engagement. Followers’ emotional exhaustion was not directly related to leaders’ emotional exhaustion over time. | The concept of crossover describes the experience of psychological states in one person affecting the experience of congruent states in another individual within the same social system. In addition to empathic crossover, two other routes to the transfer of emotions have been suggested. First, the relationship between mental states of different individuals could be spurious, particularly in an organizational setting, in which all parties are subjected to similar work demands. Second, emotional contagion could play a role. |
| 21 | Pacewicz et al. (2025) | United States | Two-wave longitudinal social network study | Adolescent baseball and softball athletes | 176 athletes from 15 teams | Youth sport teams | Teammate–teammate | Exposure to teammates’ global burnout through communication predicted athletes’ later global burnout after controlling for baseline burnout; exposure to closest friends’ burnout was not significant, and loneliness did not moderate the relationship. | Supports burnout contagion as an interpersonal, communication-based process and identifies communication exposure as a potential transmission pathway. It also suggests that contagion may depend on the structure of interpersonal exposure rather than relationship closeness alone. |
| 22 | Wörtler et al. (2026) | Netherlands | Two-wave longitudinal network study; latent change score modelling | Employees | 1,541 employees | Organizational collaboration networks | Collaborating employees / coworkers | Emotional exhaustion showed an indirect crossover effect through increased hindrance stressors, particularly among employees with shorter organizational tenure. | Emotional exhaustion may cross over among employees within collaboration networks through an indirect process in which coworkers’ exhaustion increases perceived hindrance stressors, such as role ambiguity and interpersonal conflict, which subsequently contribute to employees’ own emotional exhaustion. Organizational tenure may shape the strength of this crossover process |
| 23 | Lam et al. (2026) | China | Three-wave time-lagged multilevel study | Hotel service employees and their immediate supervisors | 445 employees nested within 83 teams | Two five-star hotels in Zhejiang Province, China | Coworker–coworker / team-level interpersonal exposure | Coworker burnout positively predicted employees’ subsequent burnout, which in turn predicted poorer work performance, supporting a burnout-contagion pathway. Coworker burnout also affected performance through a distinct work-effort signalling pathway under specific conditions. | Supports burnout contagion as a distinct interpersonal transmission process while distinguishing it from social-information effects of coworker burnout that can influence behaviour without transmission of burnout itself. |

**Supplementary Table 2. Preliminary Measurement Framework and Testable Propositions for Burnout Crossover Contagion**

| **Conceptual Component** | **Role in the Concept** | **Preliminary Measurement Indicators** | **Suggested Measures or Data Sources** | **Testable Proposition** |
| --- | --- | --- | --- | --- |
| Shared occupational stressors | Antecedent | Workload; staffing instability; shiftwork/night work; insufficient recovery; organizational support. | Staffing/rostering records; workload indicators; validated work-demand, recovery, and organizational-support measures. | P1. Shared occupational stressors may increase burnout vulnerability, but crossover effects should remain detectable after these stressors are accounted for. |
| Transmission-related conditions | Antecedent | Interaction frequency; shared shifts/shift overlap; clinical handoffs; task interdependence; relationship quality and mutual feedback. | Duty rosters; handoff/workflow records; dyadic exposure measures; social-network data. | P2. Greater interpersonal exposure may be associated with stronger subsequent burnout crossover, particularly under relational conditions that increase interpersonal receptivity. |
| Individual characteristics | Antecedent | Emotional susceptibility; self-efficacy; occupational characteristics. | Validated emotional-susceptibility and self-efficacy measures; occupational characteristics; baseline burnout. | P3. Individual characteristics may shape susceptibility to burnout crossover contagion, such that similar exposure may produce different recipient responses. |
| Interpersonal interaction | Core defining attribute | Communication; observation; feedback; repeated interpersonal exposure. | Dyadic questionnaires; interaction diaries; observational or social-network data. | P4. More frequent or intensive interpersonal interaction may increase opportunities for subsequent burnout crossover. |
| Direct and indirect transmission | Core defining attribute | Direct emotional/interpersonal processes; indirect relational/resource-related processes. | Linked source–recipient burnout assessments; repeated process measures; diaries or qualitative data. | P5. Associations between source and recipient burnout may be partly explained by direct or indirect interpersonal transmission processes. |
| Synchronization of burnout manifestations | Core defining attribute | Increasing similarity over time in emotional exhaustion, cynicism/depersonalization, and reduced professional efficacy. | Repeated dyadic/team burnout assessments; multilevel, latent-change, or social-network convergence indices. | P6. Repeated interpersonal exposure may be associated with increasing similarity in burnout manifestations among interacting individuals over time. |
| Individual and organizational consequences | Consequence | Psychological well-being; occupational attitudes; team-level burnout burden; organizational performance. Potential nursing-specific downstream implications: team functioning, care quality, and patient safety. | Validated individual outcomes; team burnout measures; performance/administrative records; exploratory care-quality and patient-safety indicators. | P7. Greater synchronization of burnout manifestations may be associated with poorer individual and organizational outcomes; broader nursing-specific outcomes require further empirical testing. |

**Note.** The framework is preliminary and hypothesis-generating. Interpersonal interaction, direct and indirect transmission, and synchronization of burnout manifestations are the core measurement domains; antecedents and consequences are supporting domains. Empirical testing should link interacting individuals and, ideally, use repeated measurement while accounting for shared occupational stressors. All proposed indicators, data sources, and propositions require empirical validation. MBI-GS, Maslach Burnout Inventory-General Survey.

**Supplementary Table 3.**  **Evidence mapping of formally included studies to conceptual elements of burnout crossover contagion**

| **Study** | **Antecedents** | | | | | | | **Defining attributes** | | | **Consequences** | | | |
| --- | --- | --- | --- | --- | --- | --- | --- | --- | --- | --- | --- | --- | --- | --- |
|  | **1** | **2** | **3** | **4** | **5** | **6** | **7** | **I** | **II** | **III** | **A** | **B** | **C** | **D** |
| Parent-Lamarche & Fernet (2020) |  |  |  |  |  | ✓ |  |  | ✓ |  | ✓ |  |  |  |
| Hakanen et al. (2014) |  |  | ✓ | ✓ |  |  | ✓ | ✓ |  |  |  |  |  |  |
| González-Morales et al. (2012) |  |  |  |  |  |  |  |  |  | ✓ |  |  | ✓ |  |
| Bakker & Schaufeli (2000) |  |  | ✓ |  | ✓ |  |  | ✓ |  | ✓ |  | ✓ |  |  |
| Westman et al. (2011) |  |  |  | ✓ |  |  |  |  |  |  |  |  | ✓ |  |
| Huang et al. (2016) |  | ✓ |  |  |  |  |  |  | ✓ |  |  |  |  |  |
| Westman & Bakker (2008) | ✓ | ✓ |  |  |  |  |  |  | ✓ |  |  |  |  |  |
| Bakker et al. (2001) |  |  |  |  | ✓ |  |  | ✓ |  | ✓ | ✓ | ✓ |  |  |
| Zimmerman (2018) |  |  |  |  |  |  |  |  |  |  |  |  |  |  |
| Bakker et al. (2005) | ✓ |  |  |  |  |  |  | ✓ |  | ✓ | ✓ | ✓ | ✓ |  |
| Meredith et al. (2020) |  |  | ✓ |  |  |  |  | ✓ |  | ✓ | ✓ |  | ✓ |  |
| Chullen (2014a) |  |  |  |  | ✓ |  |  | ✓ | ✓ | ✓ | ✓ | ✓ |  |  |
| Chullen (2014b) |  |  |  |  |  |  |  | ✓ | ✓ |  |  | ✓ |  |  |

**SUPPLEMENTARY TABLE 3 (Continued)**

| **Study** | **Antecedents** | | | | | | | **Defining attributes** | | | **Consequences** | | | |
| --- | --- | --- | --- | --- | --- | --- | --- | --- | --- | --- | --- | --- | --- | --- |
|  | **1** | **2** | **3** | **4** | **5** | **6** | **7** | **I** | **II** | **III** | **A** | **B** | **C** | **D** |
| Kim et al. (2017) |  | ✓ |  |  |  |  |  |  |  |  |  |  | ✓ |  |
| Vande Griek (2019) |  |  | ✓ |  |  |  |  | ✓ |  |  |  |  |  |  |
| Järvinen (2021) | ✓ | ✓ |  |  |  |  |  |  |  |  |  |  |  |  |
| Bakker et al. (2007) |  |  |  |  |  |  | ✓ |  | ✓ | ✓ | ✓ | ✓ |  |  |
| Dong Fuping (2017) | ✓ |  |  |  |  |  |  |  |  | ✓ |  |  |  |  |
| Westman & Etzion (1999) |  |  |  |  |  |  |  |  |  |  |  |  |  |  |
| Wirtz et al. (2016) |  |  |  |  |  | ✓ |  |  | ✓ |  |  |  |  |  |
| Pacewicz et al. (2025) |  |  | ✓ |  |  |  |  | ✓ |  | ✓ | ✓ |  |  |  |
| Wörtler et al. (2026) |  |  |  |  |  |  | ✓ | ✓ | ✓ |  |  |  |  |  |
| Lam et al. (2026) |  |  |  |  |  |  |  |  |  |  |  |  |  | ✓ |

**Note.** ✓ indicates that the included study provides direct empirical evidence or explicit conceptual/theoretical support for the corresponding element. Blank cells indicate that the study was not used as direct support for that element; some formally included studies contributed instead to concept use, conceptual boundaries, or negative-case interpretation.

**Antecedents:** 1, High-pressure work environment; 2, Limited job resources; 3, High interaction frequency; 4, Relationship quality and mutual feedback; 5, Emotional susceptibility; 6, Self-efficacy; 7, Occupational characteristics.

**Defining attributes:** I, Interpersonal interaction; II, Direct and indirect transmission; III, Synchronization of burnout manifestations.

**Consequences:** A, Impaired psychological well-being; B, Negative occupational attitudes; C, Greater team-level burnout burden; D, Declining organizational performance.

*For consequence D, the directly measured outcome in Lam et al. (2026) was employee work performance; organizational performance is retained as a cautious higher-level downstream interpretation requiring further validation.*

1.Parent-Lamarche, A., & Fernet, C. (2020). The role of employee self-efficacy in top-down burnout crossover: A longitudinal study. Journal of Occupational and Environmental Medicine, 62(10), 803–809. doi: 10.1097/JOM.0000000000001924.

2.Hakanen, J. J., Perhoniemi, R., & Bakker, A. B. (2014). Crossover of exhaustion between dentists and dental nurses. Stress and Health, 30(2), 110–121. doi: 10.1002/smi.2498.

3.González-Morales, M. G., Peiró, J. M., Rodríguez, I., & Bliese, P. D. (2012). Perceived collective burnout: A multilevel explanation of burnout. Anxiety, Stress, & Coping, 25(1), 43–61. doi: 10.1080/10615806.2010.542808.

4.Bakker, A. B., & Schaufeli, W. B. (2000). Burnout contagion processes among teachers. Journal of Applied Social Psychology, 30(11), 2289–2308. doi: 10.1111/j.1559-1816.2000.tb02437.x.

5.Westman, M., Bakker, A. B., Roziner, I., & Sonnentag, S. (2011). Crossover of job demands and emotional exhaustion within teams: A longitudinal multilevel study. Anxiety, Stress, & Coping, 24(5), 561–577. doi: 10.1080/10615806.2011.558191.

6.Huang, J., Wang, Y., Wu, G., & You, X. (2016). Crossover of burnout from leaders to followers: A longitudinal study. European Journal of Work and Organizational Psychology, 25(6), 849–861. doi: 10.1080/1359432X.2016.1167682.

7.Westman, M., & Bakker, A. B. (2008). Crossover of burnout among health care professionals. In J. R. B. Halbesleben (Ed.), Handbook of stress and burnout in health care (pp. 111–125). Nova Science Publishers.

8.Bakker, A. B., Schaufeli, W. B., Sixma, H. J., & Bosveld, W. (2001). Burnout contagion among general practitioners. Journal of Social and Clinical Psychology, 20(1), 82–98. doi: 10.1521/jscp.20.1.82.22251.

9.Zimmerman, A. S. (2018). Expanding the scope of teacher education in an attempt to prevent burnout contagion. Mid-Western Educational Researcher, 30(3), Article 5.

10.Bakker, A. B., Le Blanc, P. M., & Schaufeli, W. B. (2005). Burnout contagion among intensive care nurses. Journal of Advanced Nursing, 51(3), 276–287. doi: 10.1111/j.1365-2648.2005.03494.x.

11.Meredith, C., Schaufeli, W., Struyve, C., Vandecandelaere, M., Gielen, S., & Kyndt, E. (2020). ‘Burnout contagion’ among teachers: A social network approach. Journal of Occupational and Organizational Psychology, 93(2), 328–352. doi: 10.1111/joop.12296.

12.Chullen, C. L. (2014a). Burnout contagion in supervisor-subordinate dyads. Business and Management Research, 3(2), 67–80. doi: 10.5430/bmr.v3n2p67.

13.Chullen, C. L. (2014b). How does supervisor burnout affect leader-member exchange? A dyadic perspective. International Business & Economics Research Journal, 13(5), 1113–1126. doi: 10.19030/iber.v13i5.8777.

14.Kim, J., Youngs, P., & Frank, K. (2017). Burnout contagion: Is it due to early career teachers’ social networks or organizational exposure? Teaching and Teacher Education, 66, 250–260. doi: 10.1016/j.tate.2017.04.017.

15.Vande Griek, O. H. (2019). The upstream and downstream spread of burnout among leaders and their subordinates [Doctoral dissertation, University of Georgia].

16.Järvinen, J. (2021). Antecedents for the crossover of teacher burnout: Individual, transactional and organizational factors [Master’s thesis, University of Helsinki].

17.Bakker, A. B., Westman, M., & Schaufeli, W. B. (2007). Crossover of burnout: An experimental design. European Journal of Work and Organizational Psychology, 16(2), 220–239. doi: 10.1080/13594320701218288.

18.董阜平. (2017). 多层回归分析下的高校特殊女教工职业倦怠传染研究——以皖北部分高校为例. 湖北经济学院学报（人文社会科学版）, 14(5), 127–129, 132.

19.Westman, M., & Etzion, D. (1999). The crossover of strain from school principals to teachers and vice versa. Journal of Occupational Health Psychology, 4(3), 269–278. doi: 10.1037/1076-8998.4.3.269.

20.Wirtz, N., Rigotti, T., Otto, K., & Loeb, C. (2016). What about the leader? Crossover of emotional exhaustion and work engagement from followers to leaders. Journal of Occupational Health Psychology, 22(1), 86–97. doi: 10.1037/ocp0000024.

21.Pacewicz, C. E., Smith, A. L., & Frank, K. (2025). Teammate communication, loneliness, and contagion of athlete burnout. Psychology of Sport and Exercise, 78, 102807. doi: 10.1016/j.psychsport.2025.102807.

22.Wörtler, B., van de Brake, H. J., & van der Vegt, G. S. (2026). Crossover of emotional exhaustion in collaboration networks: The roles of hindrance stressors and organisational tenure. Work & Stress, 40(2), 125–154. doi: 10.1080/02678373.2025.2551498.

23.Lam, C. K., Huang, X., Fu, J. H. Y., Lam, W., & Chen, Z. (2026). Burnout as social information: How coworker burnout level and coworker burnout consistency interact with mastery goal orientation to affect work performance. Stress and Health, 42(4), e70204. doi: 10.1002/smi.70204
